# Analysis of Nucleolar Protein Dynamics Reveals the Nuclear Degradation of Ribosomal Proteins

**DOI:** 10.1016/j.cub.2007.03.064

**Published:** 2007-05-01

**Authors:** Yun Wah Lam, Angus I. Lamond, Matthias Mann, Jens S. Andersen

**Affiliations:** 1 Division of Gene Regulation and Expression, Wellcome Trust Biocentre, College of Life Sciences, University of Dundee, Dow Street, Dundee DD1 5EH, United Kingdom; 2 Proteomics and Signal Transduction, Max Planck Institute for Biochemistry, Am Klopferspitz 18, D-82152 Martinsried, Germany; 3 Center for Experimental BioInformatics, University of Southern Denmark, Campusvej 55, DK-5230 Odense, Denmark

**Keywords:** PROTEINS, CELLBIO

## Abstract

**Background:**

The nucleolus is a subnuclear organelle in which rRNAs are transcribed, processed, and assembled with ribosomal proteins into ribosome subunits. Mass spectrometry combined with pulsed incorporation of stable isotopes of arginine and lysine was used to perform a quantitative and unbiased global analysis of the rates at which newly synthesized, endogenous proteins appear within mammalian nucleoli.

**Results:**

Newly synthesized ribosomal proteins accumulated in nucleoli more quickly than other nucleolar components. Studies involving time-lapse fluorescence microscopy of stable HeLa cell lines expressing fluorescent-protein-tagged nucleolar factors also showed that ribosomal proteins accumulate more quickly than other components. Photobleaching and mass-spectrometry experiments suggest that only a subset of newly synthesized ribosomal proteins are assembled into ribosomes and exported to the cytoplasm. Inhibition of the proteasome caused an accumulation of ribosomal proteins in the nucleus but not in the cytoplasm. Inhibition of rRNA transcription prior to proteasomal inhibition further increased the accumulation of ribosomal proteins in the nucleoplasm.

**Conclusions:**

Ribosomal proteins are expressed at high levels beyond that required for the typical rate of ribosome-subunit production and accumulate in the nucleolus more quickly than all other nucleolar components. This is balanced by continual degradation of unassembled ribosomal proteins in the nucleoplasm, thereby providing a mechanism for mammalian cells to ensure that ribosomal protein levels are never rate limiting for the efficient assembly of ribosome subunits. The dual time-lapse strategy used in this study, combining proteomics and imaging, provides a powerful approach for the quantitative analysis of the flux of newly synthesized proteins through a cell organelle.

## Introduction

Ribosomes play a key role in gene expression, and their production is tightly linked with mechanisms for cellular-growth control (reviewed by [1]). Ribosome production is a major metabolic activity in a cell; e.g., a HeLa cell can produce up to 7500 ribosomes per minute [2]. The eukaryotic ribosome is composed of two RNA-protein subunits (60S and 40S) comprising four ribosomal RNA (rRNA) species and up to 80 ribosomal proteins (rproteins) [3]. Ribosome subunits are assembled in the nucleolus, which forms around the tandemly repeated rRNA gene clusters. rRNA genes are transcribed by RNA polymerase I, and the pre-rRNA transcripts are cleaved, modified, and assembled with rproteins to form the respective 40S and 60S ribosomal subunits. In budding yeast, both genetic and biochemical analyses have identified many of the ribosome cleavage and maturation factors (reviewed by [4]). This showed that assembly and export of the respective small (40S) and large (60S) subunits occurs predominantly via independent nucleolar maturation pathways.

The genes encoding rproteins are transcribed in the nucleoplasm by RNA polymerase II, and the rprotein mRNAs are exported to the cytoplasm and translated. Newly synthesized rproteins are imported into the nucleus, concentrate in the nucleolus, and assemble with rRNAs to form ribosome subunits. Therefore, efficient production of ribosome subunits requires the coordination of rRNA and rprotein transcription by different RNA polymerases, rprotein mRNA export and translation, and rprotein import and nucleolar targeting, as well as rRNA processing, modification, and protein assembly. The mechanisms controlling these events are still not well understood, particularly in mammalian cells.

Recently, advances in mass spectrometry (MS)-based proteomics and technologies for live-cell fluorescence imaging have revolutionized the study of protein dynamics. For example, hundreds of proteins can be identified in parallel with sensitive mass spectrometers for detection and analysis of individual peptides in complex protein mixtures. Quantitative proteomic technologies, such as SILAC [5] and iTRAQ [6], measure the relative abundance of large numbers of proteins in a single experiment. SILAC MS was recently used for analysis of the changes in relative protein levels in the nucleolar proteome when rRNA transcription was inhibited [7]. SILAC can also directly measure protein turnover [8]. In parallel, new fluorescent-protein tags have enabled quantitative measurements of protein dynamics in living cells with fluorescence microscopy. Both time-lapse studies and photobleaching techniques can be used at the single-cell level to perform repeated observations of protein localization and movements in response to specific cellular stimuli or perturbations (see [9] for review).

In this study, we have used a dual strategy that combines the power of both quantitative MS and live-cell fluorescence imaging to investigate the dynamic behavior of nucleolar proteins in mammalian cells. Our data have uncovered a mechanism that controls the participation of rproteins in ribosome-subunit assembly.

## Results

### The HeLa^RPL27-GFP^ Stable Cell Line Provides a Tool for Studying Ribosomal Biogenesis

To study the flux of mammalian rproteins, we established a HeLa cell line stably expressing ribosomal large-subunit protein L27 fused with green fluorescent protein (GFP) at its carboxy terminus (RPL27-GFP). The steady-state GFP fluorescence signal in HeLa^RPL27-GFP^ cells is predominantly in the nucleolus and cytoplasm (Figure S1A in the Supplemental Data online), consistent with other ribosomal proteins [10, 11]. The subcellular distribution and intensity of RPL27-GFP fluorescence was stable in this cell line for at least 100 passages. FACS analysis of both HeLa^RPL27-GFP^ and parental HeLa cells showed a similar distribution of cell-cycle stages (Figure S1B) and growth kinetics (Figure S1C), with no sign of apoptosis or slowed growth as a result of GFP expression.

**Figure 1:**
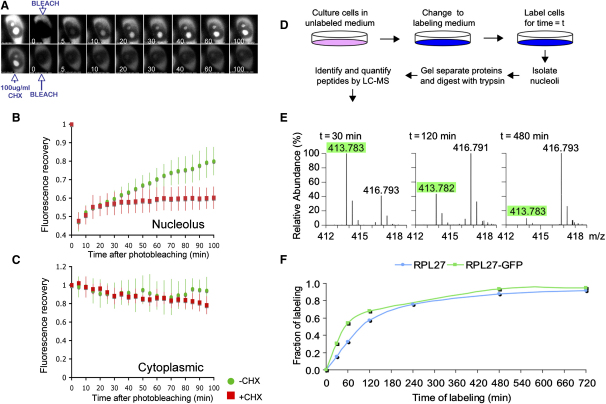
Measurement of the Rate of Nucleolar Import of RPL27-GFP by Quantitative Microscopy and Proteomics (A) The nuclear GFP fluorescence of HeLa^RPL27-GFP^ cells was photobleached, and the recovery of fluorescence in the nucleolus and cytoplasm was monitored in the absence (upper panels) and presence (bottom panels) of cycloheximide. (B) Quantitation of the relative fluorescence recovery in the nucleus after photobleaching (average ± SD of three cells) in the absence (green) and presence (red) of cycloheximide. (C) Quantitation of the relative fluorescence recovery in the cytoplasm after photobleaching (average ± SD of three cells) in the absence (green) and presence (red) of cycloheximide. (D) Design of pulse SILAC experiments. HeLa^RPL27-GFP^ cells were grown in unlabeled SILAC medium (^12^C_6_ arginine and ^12^C_6_ lysine). Experiments that were time course labeled were initiated by replacement of unlabeled medium with labeling medium (^13^C_6_ arginine and ^13^C_6_ lysine). Cells were labeled for different lengths of time, and this was followed by isolation of nucleoli. (E) Spectra of the peptide VVLVLAGR from RPL27-GFP, which illustrate the increasing incorporation of ^13^C_6_ arginine at m/z 416.79, corresponding to newly synthesized protein relative to the old pool of protein represented by the ^12^C_6_ arginine peptide signal at m/z 413.78. (F) Profiles showing increase in nucleoli of the fraction of newly synthesized RPL27 and RPL27-GFP over time were determined from the ^13^C_6_/^12^C_6_ isotope ratio from multiple peptides.

RPL27-GFP is detected as a single band of the expected size (45kDa; Figure S1D). Additional fractionation and western-blotting experiments confirmed that RPL27-GFP was in both the nucleus and cytoplasm, with most of the nuclear RPL27-GFP in the nucleolus (Figure S1D). Antibodies specific for nucleophosmin, α-tubulin, and lamin A/C were used so that the purity of the respective fractions could be confirmed (Figure S1D). Fractionation of cytoplasmic ribosomes showed that RPL27-GFP was incorporated into 60S large ribosomal subunits, 80S ribosomes, and polysomes, indicating that RPL27-GFP is a component of functional ribosomes (Figure S1E). It was not found in 40S small ribosomal subunits. For a negative control, ribosomes and polysomes were isolated from HeLa cells stably expressing free GFP alone [12], and this showed that free GFP was not incorporated into ribosome subunits, 80S ribosomes, or polysomes (Figure 1E). Taken together, the data confirm that we have established a validated GFP-marker cell line for RPL27.

### Newly Synthesized RPL27-GFP Is Rapidly Imported into the Nucleolus

The kinetics of influx of RPL27-GFP into the nucleus was analyzed by fluorescence microscopy. GFP fluorescence in five randomly selected HeLa^RPL27-GFP^ nuclei was photobleached, and nucleolar fluorescence was measured over a 2 hr time course (Figure 1A). Photobleaching did not prevent cell growth or viability because cells survived for at least 24 hr after bleaching and completed mitosis (see below and other data not shown). GFP fluorescence in the nucleoli of photobleached cells recovered from ∼45% to ∼80% of the prebleached level within 100 min, with little or no change in cytoplasmic GFP fluorescence during the same period (Figures 1B and 1C). Because the photobleaching of GFP is irreversible (see [13] for review), the net increase of GFP fluorescence should result from de novo translation of RPL27-GFP. To confirm this, we followed the recovery of nucleolar GFP after inhibiting protein synthesis with cycloheximide; recovery was minimal as a result (Figure 1B). We conclude that newly synthesized rproteins do not accumulate in the cytoplasm but instead are rapidly imported into the nucleolus. The data show that a large fraction of nucleolar RPL27 corresponds to protein synthesized in the previous 2 hr.

Next, nucleolar import of the newly synthesized RPL27 was investigated by SILAC MS. HeLa^RPL27-GFP^ cells were pulsed labeled with both ^13^C_6_ arginine and ^13^C_6_ lysine for 1–12 hr, and this was followed by isolation of nucleoli and MS analysis (Figure 1D). The isotope-incorporation rates were determined from the mass spectra of arginine or lysine containing tryptic peptides (Figure 1E). Importantly, separation of endogenous RPL27 and the RPL27-GFP fusion protein by 1D-PAGE enabled independent measurements of isotope ratios for the tagged and untagged proteins. Temporal profiles showed that ∼50% nucleolar labeling was observed for both tagged and untagged RPL27 within 2 hr (Figure 1F), confirming the rapid nucleolar import of newly synthesized rproteins detected by live-cell imaging (Figure 1B). Although we cannot exclude that the GFP tag on RPL27 has a small effect on the rate of its nucleolar import or on its assembly into ribosome subunits, the ability to quantitatively evaluate both the tagged and endogenous forms of RPL27 in the same experiment demonstrates that the populations of tagged and untagged proteins behave similarly.

### Ribosomal Proteins Accumulate in Nucleoli More Quickly than Other Nucleolar Proteins

**Figure 2:**
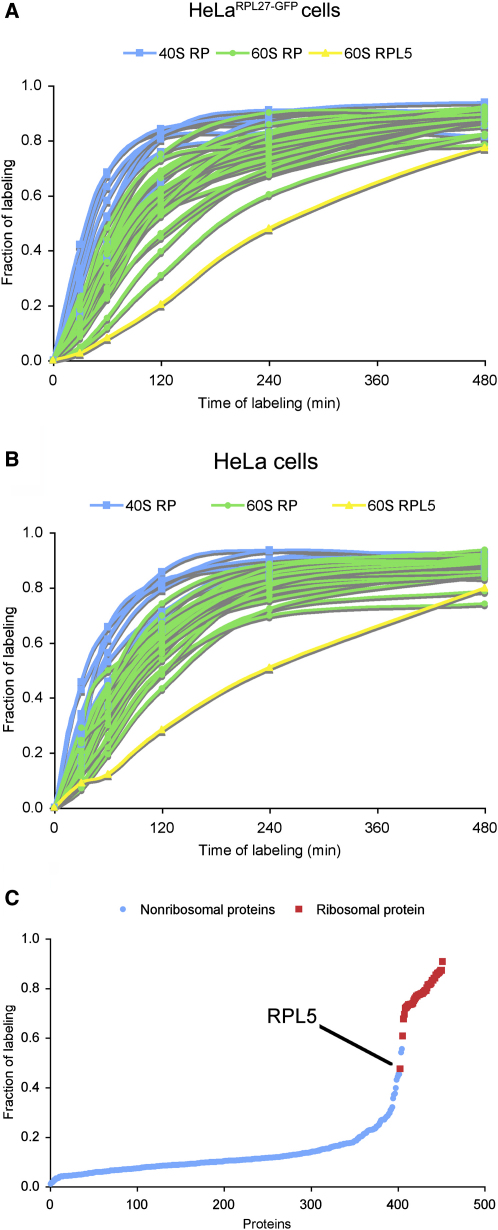
Global Analysis of Nucleolar-Protein Import by Quantitative Mass Spectrometry (A and B) Profiles of the fraction of labeling of rproteins measured from HeLa^RPL27-GFP^ cells (A) and from HeLa cells (B) determined as described in Figure 1D. The profiles are color coded in order to emphasize the distinct profiles of RPL5 (yellow) and the differences between 40S (blue) and 60S (green) rproteins. (C) Fraction of newly synthesized nucleolar rproteins (red) and non-rproteins (blue), quantified from nucleoli isolated from HeLa^RPL27-GFP^ cells labeled for 240 min.

Next, we compared the rates at which different newly synthesized proteins appear within nucleoli (Figure 2). All rproteins exhibited very fast nucleolar-incorporation rates, with typically 40%–80% of their nucleolar content corresponding to protein synthesized within the previous 2 hr (Figure 2A). The nucleolar-incorporation kinetics of rproteins in HeLa^RPL27-GFP^ and parental HeLa cells were almost identical (Figure 2A), indicating that the expression of GFP-tagged RPL27 had little or no effect on the nucleolar targeting of either RPL27 itself or other rproteins.

The rproteins accumulated more quickly than other nucleolar proteins (Figure 2C). After 4 hr of labeling, the majority of nucleolar proteins showed less than 20% isotope incorporation, whereas most rproteins showed more than 70% incorporation (Figure 2C and Table S1). Most of the 40S rproteins accumulated more quickly in nucleoli than the 60S rproteins (Figure 2A), consistent with different rates of assembly and export of the small and large ribosomal subunits. Interestingly, RPL5, which binds 5S rRNA, accumulated in nucleoli less quickly than other rproteins (Figure 2C), suggesting it has a slower import pathway.

**Figure 3:**
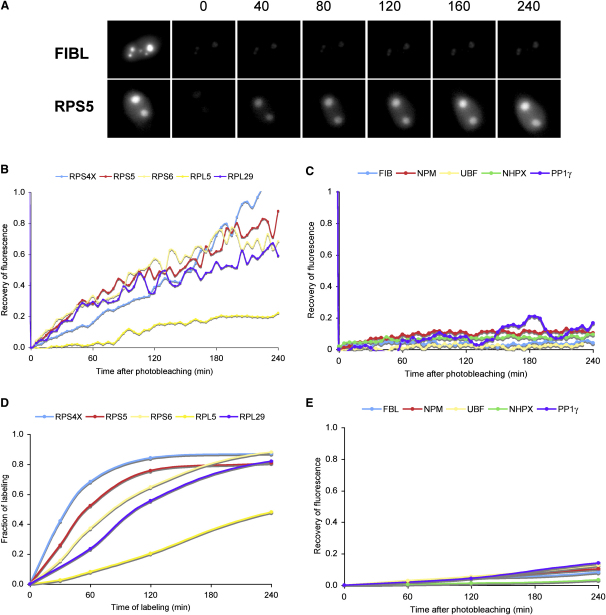
Rates of Synthesis and Import of Nucleolar Proteins (A) Fluorescence-microscopy analysis of cells expressing a GFP-tagged nonribosomal protein (fibrillarin, upper panels) and GFP-tagged ribosomal protein (RPS5, bottom panels) after whole-cell photobleaching. (B) Quantitation of the relative fluorescence recovery in the nucleoli of the photobleached cells expressing GFP-tagged ribosomal proteins. (C) Quantitation of the relative fluorescence recovery in the nucleoli of the photobleached cells expressing GFP-tagged nonribosomal proteins. (D) MS profiles of the fraction of labeling of the endogenous counterpart of proteins in (B) measured by quantitative mass spectrometry. (E) MS profiles of the fraction of labeling of the endogenous counterpart of proteins in (C) measured by quantitative mass spectrometry.

To confirm the pulse SILAC data, we analyzed nucleolar-accumulation rates by using time-lapse fluorescence microscopy, which avoids cell fractionation and isotope labeling. Ten HeLa stable cell lines were analyzed, five expressing FP-tagged rproteins (i.e., RPS6, RPS4X, RPS3, RPL5, and RPL29) and five stably expressing FP-tagged non-ribosomal nucleolar proteins (i.e., FBL, NPM, UBF, NHPX, and PP1γ). To measure their respective rates of synthesis and nucleolar targeting, we uniformly photobleached entire cells and measured recovery of nucleolar fluorescence (Figure 3). Recovery of fluorescence after photobleaching was abolished by pretreatment of cells with cycloheximide (Figure S2) and the rate of recovery was independent of the laser power used (Figure S3). Consistent with the proteomics data, the recovery rates of nucleolar GFP signals in each of the five FP-tagged rprotein cell lines (Figure 3B) were faster than recovery rates in each of the five cell lines expressing other types of FP-tagged nucleolar proteins (Figure 3C). Notably, recovery of RPL5-GFP was slower than that of other FP-tagged rproteins, consistent with the MS result. Combining the MS and microscopy data (Figure 3, compare Figures 3B and 3D with Figures 3C and 3E), we conclude that most rproteins accumulate in nucleoli more quickly than other nucleolar proteins.

### Differential Rates of Nuclear Import and Export of RPL27-GFP

**Figure 4:**
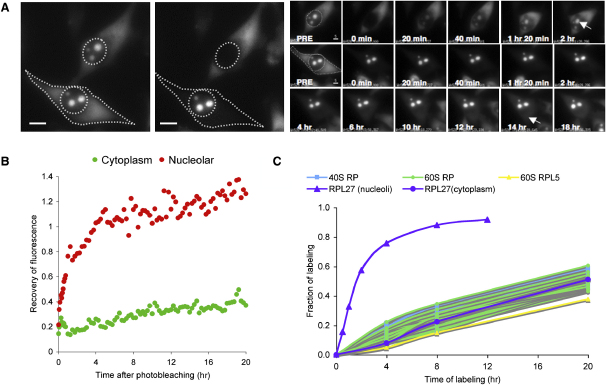
Comparing Rates of Nucleolar Import and Export of RPL27-GFP (A) Two daughter HeLa^RPL27-GFP^ cells from mitosis were chosen. The GFP fluorescence in the nucleus of the upper cell and the fluorescence in the cytoplasm of the lower cell were photobleached. The two photobleached cells were imaged every 5 min for the next 20 hr, and the fluorescence intensity in the nucleoli and cytoplasm was measured. Scale bars represent 5 μm. (B) Quantitation of the nucleolar (red) and cytoplasmic (green) fluorescence recovery. All data were expressed relative to the fluorescence intensities before photobleaching. (C) HeLa cells were labeled with ^13^C_6_^15^N_4_-arginine and ^13^C_6_^15^N_2_-lysine SILAC medium for 4, 8, and 20 hr (see Figures 1 and 2). Cytoplasmic ribosomes were purified and analyzed by LC-MS. Profiles of the fraction of labeling of rproteins were determined as described in the legend to Figure 2. The profile of nucleolar RPL27 is included as a reference.

To compare the rates of nuclear import and export of RPL27-GFP, we selected daughter HeLa^RPL27-GFP^ cells post mitosis (ensuring that both cells were at the same cell-cycle stage) and performed photobleaching to selectively remove either the nuclear or cytoplasmic GFP fluorescence (Figure 4A). Recovery of nuclear fluorescence predominantly indicated the nuclear-import rate of newly synthesized RPL27-GFP, whereas recovery of cytoplasmic fluorescence predominantly indicated the rate of export of RPL27-GFP, probably as a component of 60S ribosome subunits. Because a minimal amount of or no RPL27-GFP (or other endogenous rproteins) could be detected as free proteins in the cytoplasm (Figure S1E) and considering that cytoplasmic fluorescence recovers slowly after whole-cell photobleaching of FP-tagged rproteins (Figure S4), we infer that newly translated rproteins are targeted rapidly to the nucleolus and thus that the cytoplasmic pool of rproteins corresponds predominantly to ribosome subunits, mature ribosomes, and polysomes. Therefore, photobleaching newly translated rproteins in the cytoplasm is likely to have minimal effect on the measurement of the rate of rprotein export from the nucleus in this assay.

The recovery of nuclear and cytoplasmic fluorescence was followed for 20 hr (Figure 4B). Approximately 80% of the nuclear GFP signal was recovered 4 hr after photobleaching, consistent with the nucleolar-accumulation rates measured previously, both by FRAP (Figure 1) and by MS (Figure 2). The nuclear fluorescence continues to increase during the subsequent 16 hr. In contrast, the cytoplasmic RPL27-GFP recovery rate was much slower, with minimal recovery after 4 hr, and ∼20%–30% GFP fluorescence recovered in ∼20 hr (Figure 4B). These data derived from HeLa cells indicate that the nuclear import and export rates of RPL27-GFP are different. A similar result was obtained when RPL27-GFP was expressed in retinal pigmented epithelial cells, which unlike HeLa cells have wild-type p53 (Figure S4).

We considered it important to validate the FRAP results and therefore used SILAC to analyze untagged, endogenous proteins in cells not exposed to photodamage. Metabolic labeling of HeLa^RPL27-GFP^ cells with ^13^C_6_ arginine and ^13^C_6_ lysine was performed, and the incorporation of newly synthesized proteins in purified cytoplasmic ribosomes was measured over 20 hr (Figure 4C). Only ∼40%–60% of the rproteins in cytoplasmic ribosomes were ^13^C labeled after 20 hr, whereas ∼50% of nucleolar rproteins are ^13^C labeled within ∼2 hr of incubation in SILAC media (Figure 2). Both MS and live-cell-imaging analyses thus demonstrate a clear difference in rates of nucleolar targeting (Figure 2) and cytoplasmic export (Figure 4) of rproteins. Interestingly, RPL5, which accumulated more slowly in nucleoli than other rproteins (Figure 2), accumulated in the cytoplasmic ribosome pool at the same rate as other rproteins (Figure 4C).

### Inhibition of Protein Degradation Causes Accumulation of Nuclear RPL27-GFP

**Figure 5:**
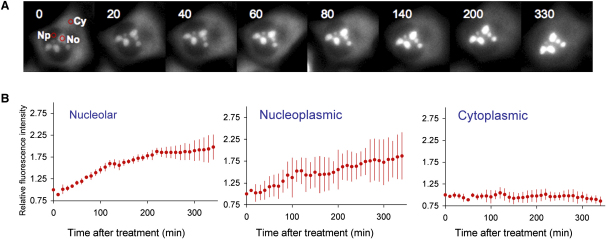
Effect of Proteasome Inhibition on RPL27-GFP (A) HeLa^RPL27-GFP^ cells were treated with epoxomicin (25 μM), and the GFP fluorescence in nucleoli (No), nucleoplasm (Np), and cytoplasm (Cy) was monitored every 5 min for 330 min. (B) Changes in RPL27-GFP fluorescence intensities in nucleoli, the nucleoplasm, and the cytoplasm (relative to the intensities before treatment) after epoxomicin treatment (average ± SD of five cells).

Although more rproteins are imported into the nucleus than are exported as stable ribosomes, we observe no consistent increase during interphase in the steady-state levels of rproteins in the nucleus. This suggests that a proportion of rproteins are degraded. To test whether rproteins are degraded in either the nucleus or cytoplasm, we analyzed the HeLa^RPL27-GFP^ stable cell line by time-lapse fluorescence microscopy in the presence of proteasome inhibitors (Figure 5).

HeLa^RPL27-GFP^ cells were incubated with epoxomicin and GFP fluorescence in five separate cells measured in the nucleolus, nucleoplasm, and cytoplasm (Figures 5A and 5B and other data not shown). RPL27-GFP accumulated in both the nucleolus and nucleoplasm with similar kinetics but did not accumulate in the cytoplasm. Remarkably, epoxomicin caused an increase of ∼80% in the nuclear RPL27-GFP level within 4 hr. This is similar to the nuclear-import rate of RPL27-GFP (Figures 1 and 2). Four additional proteasome inhibitors (i.e., lactacystin, ALLN, protease inhibitor 2, and MG132) also caused a clear increase in nuclear and nucleolar but not in cytoplasmic levels of RPL27-GFP, indicating this is likely to be a specific effect of blocking protein degradation (Figure S5). No change in GFP fluorescence was observed when cells were exposed to the same concentration of the solvent DMSO (Figure S5A). Proteasome inhibitors also did not change the levels of other nucleolar proteins, e.g., fibrillarin (Figure S5A).

On the basis of the combination of imaging (this study) and SILAC MS [7] data, we conclude that a significant fraction of the rproteins imported into the nucleus is degraded prior to export. In contrast, the cytoplasmic pool of rproteins is relatively stable and shows minimal degradation over the 5–6 hr time course of these experiments.

### Nuclear RPL27 Shuttles Continually In and Out of the Nucleolus

**Figure 6:**
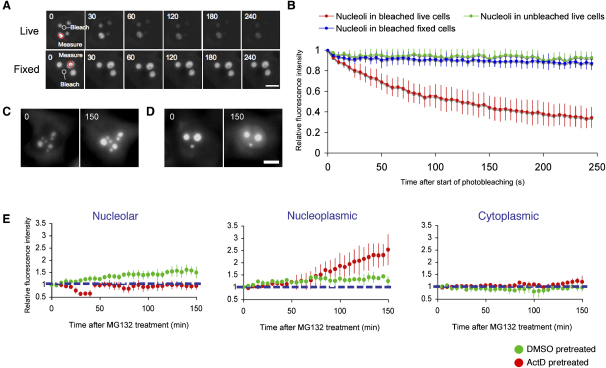
Intranuclear Shuttling of RPL27-GFP (A) Small nucleoplasmic regions (shown as white circles) in live or paraformaldehyde-fixed HeLa^RPL27-GFP^ cells were photobleached every 4 s for 50 times. The cells were imaged immediately after each bleaching event, and GFP fluorescence in the nucleoli (red circles) was measured. The scale bars represents 5 μm. (B) Quantitation of nucleolar RPL27-GFP fluorescence relative to prebleach levels in live HeLa^RPL27-GFP^ cells (red), unbleached neighboring HeLa^RPL27-GFP^ cells in the same experiment (green), and paraformaldehyde-fixed HeLa^RPL27-GFP^ cells (blue). Average ± SD from five cells in each category. (C and D) Effect of prior treatment with actinomycin D on the response of HeLa^RPL27-GFP^ cells to proteasome inhibitor MG132. HeLa^RPL27-GFP^ cells were treated with either 1/5000 (v/v) DMSO (C) or 0.5 μg/ml actinomycin D (stock solution: 2.5mg/ml in DMSO) (D) for 1 hr before the addition of 25 μM MG132. In both cases, the same cell was imaged before (left panel) and 150 min after (right panel) MG132 treatment. The scale bar represents 5 μm. (E) Changes in RPL27-GFP fluorescence intensities in nucleoli, the nucleoplasm, and the cytoplasm (relative to the intensities before treatment. Average ± SD of five cells). Green represents cells pretreated with DMSO before MG132 treatment. Red represents cells pretreated with actinomycin D before MG132 treatment.

Nuclear-proteasome activity has been detected in the nucleoplasm but not in nucleoli [14]. To test whether nucleolar rproteins are either static or continually shuttling between the nucleolus and nucleoplasm, where they would be exposed to nucleoplasmic proteasomes, we performed fluorescence loss in photobleaching (FLIP) experiments on the HeLa^RPL27-GFP^ cells (see [13] for review). A region in the nucleoplasm of HeLa^RPL27-GFP^ cells was repeatedly photobleached, thus resulting in a rapid depletion of GFP signal from nucleoli (Figures 6A and 6B). A similar experiment performed on a fixed cell did not result in any change in the nucleolar GFP fluorescence (Figures 6A and 6B). The rapid decrease of nucleolar RPL27-GFP fluorescence (∼80% depletion in 4 min, Figure 6B) was too fast to be caused simply by the bleaching of newly imported RPL27-GFP. We conclude that RPL27-GFP, in common with many other nucleolar proteins [15], can cycle continually between the nucleolus and nucleoplasm, thus accounting for the accessibility of nucleolar rproteins to nucleoplasmic proteasomes.

### Both rRNA Synthesis and Proteasome Activity Affect Nuclear rprotein Localization

The nucleolar accumulation of rproteins is likely to be due, at least in part, to their binding to rRNA transcripts because SILAC MS has shown that rprotein levels in purified nucleoli decrease when rRNA transcription is inhibited [7]. To test whether the steady-state distribution of rproteins in the nucleus reflects the interplay between binding to rRNA in the nucleolus and degradation by the proteasome in the nucleoplasm, we compared the effect of proteasome inhibitors on the nuclear distribution of rproteins in cells with or without prior inhibition of rRNA synthesis (Figures 6C and 6D). There is a clear enhancement in the nucleoplasmic accumulation of RPL27-GFP, when inhibition of the proteasome with MG132 is preceded by blocking rRNA transcription with actinomycin D (Figure 6e). By contrast, both nucleolar and cytoplasmic RPL27-GFP levels showed little or no change when the cells were treated with actinomycin D before MG132 treatment (Figure 6E).

### rRNA Synthesis Affects rprotein Mobility in the Nucleus

**Figure 7:**
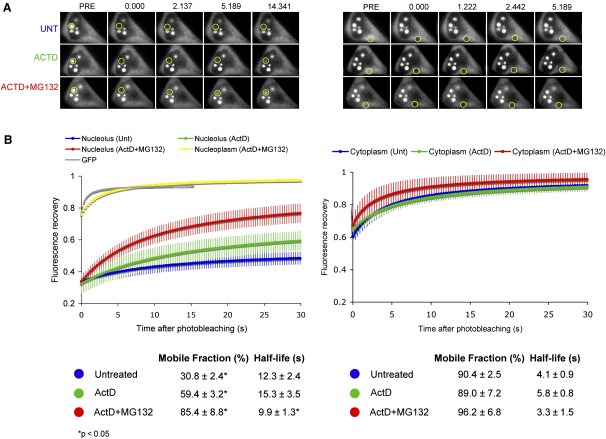
FRAP of Nucleolar and Cytoplasmic RPL27-GFP after Drug Treatment (A) Small regions in the nucleolus (left panels) and the cytoplasm (right panels) of a HeLa^RPL27-GFP^ cell were photobleached, and the recovery of fluorescence was measured. A total of 0.5 μg/ml of actinomycin D was then added to the cells and after an incubation of 1 hr; the same regions of the same cell were photobleached again and measured. A total of 25 μM MG132 was then added, and after an additional incubation of 2 hr, the same regions of these cells were photobleached and measured again. (B) The kinetics of relative fluorescence recovery of nucleolar (left graph) and cytoplasmic (right graph) RPL27-GFP in untreated (blue), actinomycin D-treated (green) and actinomycin D-plus-MG132-treated (red) cells. Each data point shows average ± SD of five cells. The relative recovery of fluorescence in the nucleoplasm of HeLa^RPL27-GFP^ treated with both actinomycin D and MG132 is shown in yellow. The relative recovery of fluorescence in the nucleoplasm of HeLa^GFP^ is shown in gray.

Next, we addressed whether the shuttling pool of nuclear rproteins correspond to either free rproteins or larger complexes, such as partially assembled ribosome subunits. To overcome technical difficulties caused by the normally low steady-state levels of nucleoplasmic rprotein, we performed FRAP experiments in cells pretreated with actinomycin D for 1 hr, and this was followed by MG132 treatment to enhance nucleoplasmic fluorescence to measurable levels (Figure 7). FRAP measurements were made separately on nucleoplasmic, nucleolar, and cytoplasmic pools of rproteins in each case. This showed a higher mobility of the rprotein population in the nucleoplasm, as compared with either the nucleolus or cytoplasm (Figure 7B). The nucleoplasmic pool of RPL27-GFP has rapid mobility, similar to free GFP. By contrast, cytoplasmic RPL27-GFP has slower mobility, consistent with its association in large ribosomal complexes (Figure 7B). Notably, the nucleolar fraction of RPL27-GFP shows the slowest mobility of all, consistent with its association with large pre-rRNA-processing complexes and intermediates of ribosome-subunit assembly. We conclude that the major component of the nucleoplasmic rprotein pool is composed of either free rprotein or small protein complexes.

Finally, we analyzed the effect of inhibiting rRNA synthesis with actinomycin D on the mobility of rprotein in the nucleolar compartment (Figure 7). This shows very low levels of recovery in the nucleolus when rRNA synthesis is active (i.e., no actinomycin D treatment), whereas the mobile fraction is clearly increased after blocking rRNA synthesis alone and increased more upon blocking both rRNA synthesis and the proteasome (Figure 7B). In contrast, neither inhibitor causes a major mobility change in the cytoplasmic rprotein fraction (Figure 7B). Control experiments showed that the recovery rates were not significantly influenced by the sequential spot-photobleaching protocol used (Figure S6). These data strongly suggest that rRNA synthesis contributes to tethering rproteins in low-mobility complexes within the nucleolus.

## Discussion

In this study, we have analyzed the synthesis and intracellular dynamics of rproteins in cultured mammalian cells by using a dual strategy combining quantitative MS and live-cell time-lapse fluorescence imaging. Both approaches produced similar results and showed that rproteins are synthesized and accumulated in nucleoli more rapidly than any other nucleolar factors. A clear difference was observed in the amount of rprotein imported into the nucleus relative to that subsequently exported to the cytoplasm as ribosome subunits. This was shown to result from the continual rprotein nucleoplasmic degradation, which could be prevented by inhibition of the proteasome. The nuclear rproteins can shuttle in and out of the nucleolus, mostly in the form of either free proteins or as small protein complexes, but are retained within nucleoli in slow-mobility complexes when rRNA synthesis is active. These observations account for the steady-state distribution of rproteins within the cell and indicate the existence of a previously unknown mechanism that may contribute to the efficient production of ribosome subunits in mammalian cells.

Here, we have utilized a “dual strategy,” combining two powerful and complementary techniques to study intracellular protein dynamics, i.e., quantitative MS-based proteomics and live-cell fluorescence microscopy. Both methods generate quantitative “time-lapse” measurements of changes in protein levels in different cellular compartments but rely upon completely different analytical procedures. Importantly, both methods can be applied in parallel for analysis of the same biological response in cultured cell lines stably expressing GFP-tagged fusion proteins, thereby facilitating a direct comparison of the measurements made with each technique. One method analyses protein mobility through fluorescence measurements on tagged proteins expressed in living cells, whereas the other uses metabolic labeling and cell fractionation to perform high throughput analysis of endogenous, untagged proteins. Thus, when two such distinct approaches generate similar results, it is more likely that the measurements obtained accurately reflect cellular responses because it is unlikely that any artifact or limitation influencing one method will also affect the other. For example, the presence of a GFP tag could influence the function, dynamic properties, localization, or stability of the resulting fusion protein and requires exposure of cells to potentially damaging external light. However, the SILAC MS approach simultaneously measures the behavior of endogenous, untagged proteins as well as tagged reporters and therefore eliminates this concern. In contrast, GFP-fusion proteins allow the analysis of the same live cells at multiple time points and avoids the requirement for metabolic labeling, cell fractionation, and protein purification prior to the quantitation of protein levels.

The advantage of this dual approach is well illustrated here by the comparison of both the GFP-tagged and endogenous forms of RPL27 in the HeLa^RPL27-GFP^ stable cell line. The use of SILAC analysis shows that the bulk populations of both forms of RPL27 behave similarly and also allows a high throughput comparison of RPL27 with other rproteins and nucleolar factors. Furthermore, we show a direct comparison of the behavior of RPL27 in both the HeLa^RPL27-GFP^ stable cell line and in the parental HeLa cell line that does not express any fusion proteins. Together with the additional detailed characterization and comparison of these cell lines, with fluorescence microscopy, FACS analysis, and protein blotting, it was possible to show with confidence that the HeLa^RPL27-GFP^ stable cell line provides a valid model for analyzing rprotein dynamics in living cells. We therefore propose that this dual MS/microscopy strategy provides a useful paradigm for the future design of cell biological experiments based upon the analysis of epitope and FP-tagged proteins.

Both the MS and fluorescence microscopy approaches showed that rproteins were rapidly synthesized and accumulated in nucleoli more quickly than any other of the nucleolar proteins detected. However, it was apparent that RPL5, although still showing rapid kinetics, was consistently slower to accumulate in nucleoli than any of the other rproteins analyzed. Interestingly, previous studies in HeLa cells have shown that RPL5, unlike other rproteins, associates with 5S rRNA in the nucleoplasm prior to its import into the nucleolus [16], and such an association may account for its delayed kinetics of nucleolar accumulation relative to other rproteins. It was notable that RPL5 showed similar dynamic behavior to all the other rproteins when the cytoplasmic pool was analyzed, further indicating that the differences in the nuclear pool relate to events prior to ribosome-subunit export.

Very few other proteins accumulate in nucleoli at a rate close to rproteins, the main exceptions being TGFβ-inducible nuclear protein 1 and p14Arf (Table S1). p14Arf is known to regulate p53 activity and to play an important role in growth-rate control (reviewed by [17]). It is possible that one or more of its functions may also regulate ribosome-subunit production, which must be tightly linked with cell-growth rate. It will be interesting in future to test whether this may be related to its rapid import kinetics into nucleoli. TGFβ-inducible nuclear protein 1 was first identified at a gene locus associated with hairy-cell leukaemia [18]. It has recently been shown to be a functional homolog of yeast Nsa2p, which is essential for ribosomal large-subunit biogenesis [19]. Interestingly, Nsa2p is found to be a short-lived protein that is quickly degraded when ribosomal biogenesis is blocked [19]. It is interesting that our present study on protein dynamics highlighted TGFβ-inducible nuclear protein 1 without prior knowledge of the data showing that its functional homolog in budding yeast was linked with the biogenesis of the large ribosomal subunit. It will be interesting to examine whether the unusual kinetic behavior of TGFβ-inducible nuclear protein 1/hNsa2p is also relevant to large-ribosome-subunit biogenesis or linked to the mechanism of hairy-cell leukemia.

The SILAC analysis also shows a clear difference between the rates of nucleolar accumulation for small and large subunit rproteins, and this is supported by analysis involving live-cell fluorescence imaging of all six stable cell lines analyzed that express GFP-tagged rproteins, including examples for both the small and large ribosome subunits. Interestingly, our previous SILAC analysis of nucleolar proteins, with steady-state rather than pulsed labeling conditions, also showed a difference between small and large subunit rproteins in terms of their response to inhibiting rRNA synthesis with actinomycin D [7]. Thus, analysis of nucleoli isolated from HeLa cells after treatment with actinomycin D showed that the relative level of rproteins decreased, with a larger decrease evident for the small-subunit proteins. Previous studies in budding yeast have shown that the small-subunit rproteins bind to the pre-rRNA earlier than large-subunit proteins and also have shown that the respective small and large subunits are matured at different rates and exported independently from the yeast nucleolus [20, 21]. Our present data are consistent with a similar difference in the rate of small- and large-ribosome-subunit formation occurring in mammalian cells.

The most unexpected finding in this study is that a significant fraction of the rproteins that are imported into the nucleus is degraded and not assembled into ribosome subunits. This is illustrated by the dramatic nuclear accumulation of all GFP-tagged rproteins analyzed by time-lapse fluorescence microscopy upon inhibition of the proteasome. In the case of the HeLa^RPL27-GFP^ stable cell line, five separate proteasome inhibitors were tested independently, and each caused a rapid increase in levels of nuclear, but not cytoplasmic, RPL27-GFP within 5 hr. Importantly, a similar rapid increase in nucleolar levels of endogenous, untagged rproteins after proteasome inhibition was also demonstrated by SILAC MS [7]. Furthermore, a high throughput mass-spectrometric identification of endogenous, ubiquitinylated proteins in human cells detected many examples of ubiquitin-conjugated rproteins, confirming that they are likely to be substrates for a proteasome-mediated degradation pathway [22]. We note that proteasome inhibitors, when used at high concentrations, have also been shown to cause changes in nucleolar morphology and to block rRNA processing in mammalian cells [23]. This suggests that the conjugation of ubiquitin to rproteins, in addition to regulating the nuclear levels of rproteins through proteasome degradation, may also have direct or indirect effects on the ribosomal-biogenesis pathway.

It is likely that nuclear rprotein degradation occurs in the nucleoplasm, rather than in nucleoli. This view is supported by recent biochemical experiments demonstrating that high levels of proteasome activity are detected in the nucleoplasm, whereas nucleoli are devoid of proteasome activity [14]. Consistent with this, previous mass-spectrometric analyses of the nucleolar proteome did not detect significant levels of proteasome components in purified nucleoli [7, 24, 25]. Although most rproteins in the nucleus accumulate in nucleoli, this is not inconsistent with degradation occurring outside in the nucleoplasm because the FLIP experiments performed in this study show that there is a continual movement of rproteins in and out of nucleoli. Therefore, rproteins will be exposed to the proteasome whenever they traffic into the nucleoplasm, and we propose this contributes to the very low level of nucleoplasmic rproteins that can accumulate at steady state (Figure S7).

On the basis of the FRAP analysis of rprotein mobility, it appears that most of the rprotein shuttling into the nucleoplasm is likely either to be free protein or to be in small complexes, rather than assembled into ribosomal subunits. We therefore infer that it is predominantly free rproteins in the nucleus that are substrates for the proteasome, whereas conversely we infer that rproteins assembled into ribosomal subunits are largely resistant to degradation. This can explain why the cytoplasmic pool of rproteins, which we detect almost exclusively associated with ribosomal subunits, mature ribosomes, and polysomes, is stable to degradation. We therefore suggest a model in which great amounts of newly translated rproteins are continually synthesized and rapidly imported into the nucleus where they shuttle between the nucleolus and nucleoplasm (Figure S7). When rRNA transcription occurs, the rproteins become tethered in slow-mobility nucleolar complexes, which presumably correlate with ribosome subunits and processing intermediates. Once ribosome-subunit assembly is completed, the RNPs are then exported to the cytoplasm where they can engage in translation. In contrast, free rproteins in excess of the requirement for ribosome-subunit production, as dictated by levels of rRNA synthesis, will continue to shuttle into the nucleoplasm where they are exposed to proteasome-mediated degradation.

We note that this model, which is derived from both MS and live-cell-imaging experiments, is compatible with much earlier studies on HeLa cells with radio-labeling procedures to study proteins that copurify with ribosomes. For example, by using cell fractionation, pulse-chase radiolabeling and 2D gel separation of proteins, Warner reported that newly synthesized rproteins are rapidly targeted to the nucleolus and the nucleoplasmic level of newly synthesized rproteins increased when rRNA synthesis was inhibited [26]. Thus, although these early studies used techniques that could not identify individual protein species, nonetheless they show that newly synthesized rproteins rapidly appear in nucleoli and also showed that rproteins are relatively unstable when rRNA transcription is inhibited [27, 28]. By contrast, cytoplasmic ribosomes are metabolically stable and have long half-lives relative to most other RNA and protein species.

Our model is also supported by observations in *Saccharomyces cerevisiae* by Rosbash and colleagues [29], who expressed an exogenous RPS51 gene tagged with lacZ, either with or without the wild-type RPS51 gene. Interestingly, they found that in the absence of endogenous RPS51, RPS51-lacZ was associated with polysomes, but in the presence of endogenous RPS51, RPS51-lacZ was not only expressed ten times less efficiently but also predominately located in the nucleus and not in polysomes. The RPS51-lacZ mRNA levels were equal in both cases. This demonstrated that rproteins can be regulated posttranslationally, with excess rproteins being retained in the nucleus. When extra copies of genes encoding RPS51 [30], RPS10, or RPL29 [31] were introduced to yeast cells, these exogenous rprotein mRNAs were translated, but the resulting rproteins were highly unstable. In the case of RPS51, the excess protein was found to be degraded within 3 min after its synthesis [29]. Reduction of RPS51 mRNA levels only modestly reduced cell growth, suggesting that yeast RPS51 may be synthesized in excess under normal conditions, and this synthesis can compensate partially for the reduction in mRNA levels [29].

In our model, the rate of rRNA synthesis is proposed as the major control point that is likely to regulate the overall rate of ribosome-subunit production. This is achieved at the expense of an apparent overproduction of rproteins. However, we suggest that this unexpected mechanism is used in mammalian cells to ensure that the critical cellular process of efficient ribosome subunit-production is never limited by the available supply of rproteins and avoids a potentially toxic accumulation of unbound, free rproteins in the nucleoplasm. We note that the high turnover of nuclear rproteins is probably not due simply to the absence of wild-type p53 in HeLa cells because expression of GFP-tagged rproteins in human retinal pigmented epithelial cells, which have a functional p53 gene, also display a similar effect (Figure S4). It will therefore be interesting to examine more closely in future the generality of the rprotein-degradation model that we have detected in mammalian cells and to explore further its regulatory implications.

## Experimental Procedures

### Cell Culture

Transfection and establishment of stable cell lines were done as described [12]. The establishment of HeLa^RPL27-GFP^, HeLa^Fibrillarin-GFP^, and HeLa^NPM-GFP^ stable cell lines was reported in [32], and the establishment of HeLa^PP1γ-GFP^ and HeLa^GFP^ in [12] and HeLa^NHPX-GFP^ was reported in [33]. All cell lines were maintained in DMEM (Invitrogen) with 10% fetal-bovine serum (Invitrogen) at 37°C and in 5% CO_2_.

### Microscopy

All imaging experiments were performed on a wide-field fluorescence microscope (DeltaVision Spectris; Applied Precision) fitted with an environmental chamber (Solent Scientific) so that temperature at 37°C was maintained and fitted with a CoolMax charge-coupled device camera (Roper Scientific). Detailed protocols for live-cell imaging are described in the Supplemental Experimental Procedures.

### Mass Spectrometry

HeLa and HeLa^RPL27-GFP^ cells were grown in SILAC medium with dialyzed serum as described [34]. Time-course-labeling experiments were initiated by the replacement of ^12^C_6_ arginine and ^12^C_6_ lysine medium with pre-equilibrated ^13^C_6_ arginine and ^13^C_6_ lysine medium for 30, 60, 120, 240, 480, and 720 min. Nucleoli were isolated directly from time-course-labeled cells as described [35]. Isolated nucleolar proteins were separated on NuPAGE 4%−12% Bis-Tris gels and cut into 11 slices, and each slice was digested with trypsin. Peptides resulting from in-gel digestion were extracted from the gel slices, desalted, concentrated on reverse-phase C18 Stage tips, and eluted into 96-well plates for automated MS analysis as described [7]. Detailed MS procedures are described in the Supplemental Experimental Procedures.

## Supplemental Data

Document S1. Additional Experimental Procedures and Six Figures

Table S1. Proteins Identified and Quantified by Mass Spectrometry from Nucleoli Isolated from RPL27-GFP HeLa cells after 240 Min of Isotope Labeling Proteins identified and quantified by mass spectrometry from nucleoli isolated from RPL27-GFP HeLa cells after 240 min of isotope labeling and plotted in Figure 2B.

Table S2. Ribosomal Proteins Identified and Quantified by Mass Spectrometry from Nucleoli Isolated from HeLaRPL27-GFP Cells and HeLa Cells after 30–720 Min of Isotope Labeling Ribosomal proteins identified and quantified by mass spectrometry from nucleoli isolated from HeLa^RPL27-GFP^ cells and HeLa cells after 30–720 min of isotope labeling. Data are plotted in Figure 2A.

## References

[1] Mayer C., Grummt I. (2006). Ribosome biogenesis and cell growth: mTOR coordinates transcription by all three classes of nuclear RNA polymerases. Oncogene.

[2] Lewis J.D., Tollervey D. (2000). Like attracts like: Getting RNA processing together in the nucleus. Science.

[3] Tate W.P., Poole E.S. (2004). The ribosome: Lifting the veil from a fascinating organelle. Bioessays.

[4] Granneman S., Baserga S.J. (2004). Ribosome biogenesis: Of knobs and RNA processing. Exp. Cell Res..

[5] Mann M. (2006). Functional and quantitative proteomics using SILAC. Nat. Rev. Mol. Cell Biol..

[6] Ross P.L., Huang Y.L.N., Marchese J.N., Williamson B., Parker K., Hattan S., Khainovski N., Pillai S., Dey S., Daniels S. (2004). Multiplexed protein quantitation in Saccharomyces cerevisiae using amine-reactive isobaric tagging reagents. Mol. Cell. Proteomics.

[7] Andersen J.S., Lam Y.W., Leung A.K., Ong S.E., Lyon C.E., Lamond A.I., Mann M. (2005). Nucleolar proteome dynamics. Nature.

[8] Doherty M.K., Whitehead C., McCormack H., Gaskell S.J., Beynon R.J. (2005). Proteome dynamics in complex organisms: Using stable isotopes to monitor individual protein turnover rates. Proteomics.

[9] Shav-Tal Y., Darzacq X., Singer R.H. (2006). Gene expression within a dynamic nuclear landscape. EMBO J..

[10] Jakel S., Gorlich D. (1998). Importin beta, transportin, RanBP5 and RanBP7 mediate nuclear import of ribosomal proteins in mammalian cells. EMBO J..

[11] Plafker S.M., Macara I.G. (2002). Ribosomal protein L12 uses a distinct nuclear import pathway mediated by importin 11. Mol. Cell. Biol..

[12] Trinkle-Mulcahy L., Andrews P.D., Wickramasinghe S., Sleeman J., Prescott A., Lam Y.W., Lyon C., Swedlow J.R., Lamond A.I. (2003). Time-lapse imaging reveals dynamic relocalization of PP1gamma throughout the mammalian cell cycle. Mol. Biol. Cell.

[13] Lippincott-Schwartz J., Altan-Bonnet N., Patterson G.H. (2003). Photobleaching and photoactivation: Following protein dynamics in living cells. Nat. Cell Biol..

[14] Rockel T.D., Stuhlmann D., von Mikecz A. (2005). Proteasomes degrade proteins in focal subdomains of the human cell nucleus. J. Cell Sci..

[15] Misteli T. (2001). The concept of self-organization in cellular architecture. J. Cell Biol..

[16] Steitz J.A., Berg C., Hendrick J.P., La Branche-Chabot H., Metspalu A., Rinke J., Yario T. (1988). A 5S rRNA/L5 complex is a precursor to ribosome assembly in mammalian cells. J. Cell Biol..

[17] Gallagher S.J., Kefford R.F., Rizos H. (2006). The ARF tumour suppressor. Int. J. Biochem. Cell Biol..

[18] Wu X., Ivanova G., Merup M., Jansson M., Stellan B., Grander D., Zabarovsky E., Gahrton G., Einhorn S. (1999). Molecular analysis of the human chromosome 5q13.3 region in patients with hairy cell leukemia and identification of tumor suppressor gene candidates. Genomics.

[19] Lebreton A., Saveanu C., Decourty L., Jacquier A., Fromont-Racine M. (2006). Nsa2 is an unstable, conserved factor required for the maturation of 27 SB pre-rRNAs. J. Biol. Chem..

[20] Nissan T.A., Bassler J., Petfalski E., Tollervey D., Hurt E. (2002). 60S pre-ribosome formation viewed from assembly in the nucleolus until export to the cytoplasm. EMBO J..

[21] Schafer T., Strauss D., Petfalski E., Tollervey D., Hurt E. (2003). The path from nucleolar 90S to cytoplasmic 40S pre-ribosomes. EMBO J..

[22] Matsumoto M., Hatakeyama S., Oyamada K., Oda Y., Nishimura T., Nakayama K.I. (2005). Large-scale analysis of the human ubiquitin-related proteome. Proteomics.

[23] Stavreva D.A., Kawasaki M., Dundr M., Koberna K., Muller W.G., Tsujimura-Takahashi T., Komatsu W., Hayano T., Isobe T., Raska I. (2006). Potential roles for ubiquitin and the proteasome during ribosome biogenesis. Mol. Cell. Biol..

[24] Andersen J.S., Lam Y.W., Leung A.K., Ong S.E., Lyon C.E., Lamond A.I., Mann M. (2002). Directed proteomic analysis of the human nucleolus. Curr. Biol..

[25] Scherl A., Coute Y., Deon C., Calle A., Kindbeiter K., Sanchez J.C., Greco A., Hochstrasser D., Diaz J.J. (2002). Functional proteomic analysis of human nucleolus. Mol. Biol. Cell.

[26] Warner J. (1979). Distribution of newly formed ribosomal proteins in HeLa cell fractions. J. Cell Biol..

[27] Warner J. (1977). In the absence of ribosomal RNA synthesis, the ribosomal proteins of HeLa cells are synthesized normally and degraded rapidly. J. Mol. Biol..

[28] Wu R.S., Kumar A., Warner J. (1971). Ribosome formation is blocked by camptothecin, a reversible inhibitor of RNA synthesis. Proc. Natl. Acad. Sci. USA.

[29] Abovich N., Gritz L., Tung L., Rosbash M. (1985). Effect of RP51 gene dosage alterations on ribosome synthesis in Saccharomyces cerevisiae. Mol. Cell. Biol..

[30] Warner J., Mitra G., Schwindinger W.F., Studeny M., Fried H.M. (1985). Saccharomyces cerevisiae coordinates accumulation of yeast ribosomal proteins by modulating mRNA splicing, translational initiation, and protein turnover. Mol. Cell. Biol..

[31] Gritz L., Abovich N., Teem J.L., Rosbash M. (1985). Posttranslational regulation and assembly into ribosomes of a Saccharomyces cerevisiae ribosomal protein-beta-galactosidase fusion. Mol. Cell. Biol..

[32] Leung A.K., Gerlich D., Miller G., Lyon C., Lam Y.W., Lleres D., Daigle N., Zomerdijk J., Ellenberg J., Lamond A.I. (2004). Quantitative kinetic analysis of nucleolar breakdown and reassembly during mitosis in live human cells. J. Cell Biol..

[33] Leung A.K., Lamond A.I. (2002). In vivo analysis of NHPX reveals a novel nucleolar localization pathway involving a transient accumulation in splicing speckles. J. Cell Biol..

[34] Ong S.E., Mann M. (2006). A practical recipe for stable isotope labeling by amino acids in cell culture (SILAC). Nature Protocols.

[35] Lam Y.W., Lamond A.I., Celis J.E. (2006). Isolation of nucleoli.

