# Supplementary material for: Analysis of Nucleolar Protein Dynamics Reveals the Nuclear Degradation of Ribosomal Proteins
Source: Curr Biol. 2007 May 1;17(9):749–60. doi: 10.1016/j.cub.2007.03.064 (PMC1885954; doi:10.1016/j.cub.2007.03.064)
Supplement: Document S1. Additional Experimental Procedures and Six Figures [file mmc1.pdf]

Supplemental Data

Analysis of Nucleolar Protein Dynamics Reveals the Nuclear Degradation of Ribosomal Proteins

Yun Wah Lam, Angus I. Lamond, Matthias Mann, and Jens S. Andersen

Supplemental Experimental Procedures

Plasmids, Antibodies and Drugs

RPS4X-GFP and RPS6-GFP were cloned by insertion of the appropriate EST sequences into the EGFP-C1 vector. RPS5-GFP and RPL29-GFP plasmids are kind gifts from Dr. Ulrike Kutay (Swiss Federal Institute of Technology, Switzerland) [S1]. The RPL5-GFP plasmid is a kind gift from Dr. Hua Lu (Oregon Health & Science University, Portland, OR) [S2]. Mouse anti-GFP was purchased from Roche, and mouse anti-lamin A/C, mouse anti-nucleophosmin, mouse anti-RPS6, and goat anti-RPL28 were purchased from Santa Cruz Biotechnology. Secondary antibodies used for western blotting were from Pierce. Cycloheximide was purchased from Sigma. Proteasome inhibitors epoxomicin, lactacystin, ALLN, protease inhibitor 2, and MG132 were from Calbiochem. Actinomycin D was from Sigma. Stock solutions were prepared by dissolution of the drugs in DMSO (except for cycloheximide that was dissolved in culture medium) at the concentrations indicated in the figure legends. In all cases, the same volume of DMSO was used as a control.

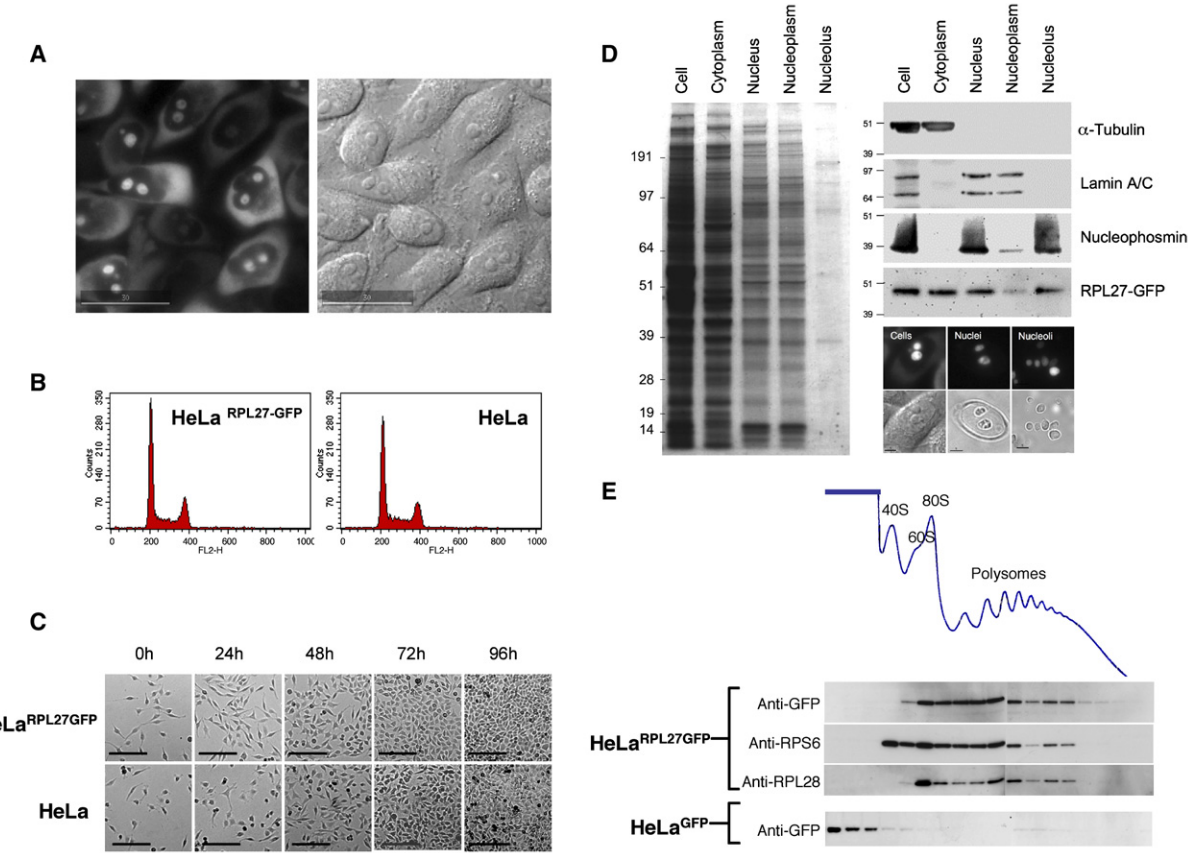

Figure S1. Characterization of the HeLa $^{RPL27-GFP}$ Stable Cell Line

(A) The GFP fluorescence (left) and cell morphology (right) of HeLa $^{RPL27-GFP}$ cells. Scale bars represent 30 μm.

(B) The cell-cycle-stage distribution of HeLa $^{RPL27-GFP}$ (left) and the parental HeLa cells (right) as measured by flow cytometry.

(C) Growth rates of HeLa $^{RPL27-GFP}$ (upper panels) and the parental HeLa (bottom panels) cells were estimated by time-lapse DIC microscopy on live cells. Equal numbers of HeLa $^{RPL27-GFP}$ and HeLa cells were seeded on chambered coverglass and observed every 24 hr for 4 days. Scale bars represent 100 μm.

(D) HeLa $^{RPL27-GFP}$ cells were separated into cytoplasmic, nuclear, nucleoplasmic, and nucleolar fractions. The left panel shows the Coomassie staining of proteins from each fraction separated by SDS-PAGE. For each lane, fractions isolated from 2×10^4 cells were used. The upper right panel shows western blotting of HeLa $^{RPL27-GFP}$ cells, and subcellular fractions (each from 2×10^4 cells) was immunolabeled with antibodies against α-tubulin, lamin A/C, nucleophosmin, and RPL27-GFP. Lower right panels show GFP fluorescence and DIC images of HeLa $^{RPL27-GFP}$ cells, nuclei and nucleoli (Scale bars represent 5 μm.).

(E) Cytoplasmic extract from HeLa $^{RPL27-GFP}$ cells was fractionated by a sucrose gradient. The presence of 40S, 60S, and 80S ribosomes and polysomes were shown by OD260 measurement and by western blotting with antibodies against endogenous RPS6 and RPL28. RPL27-GFP was detected by anti-GFP. In a separate experiment, HeLa ^{GFP} cells were fractionated with an identical protocol, and the fractions were labeled by anti-GFP (bottom panel).

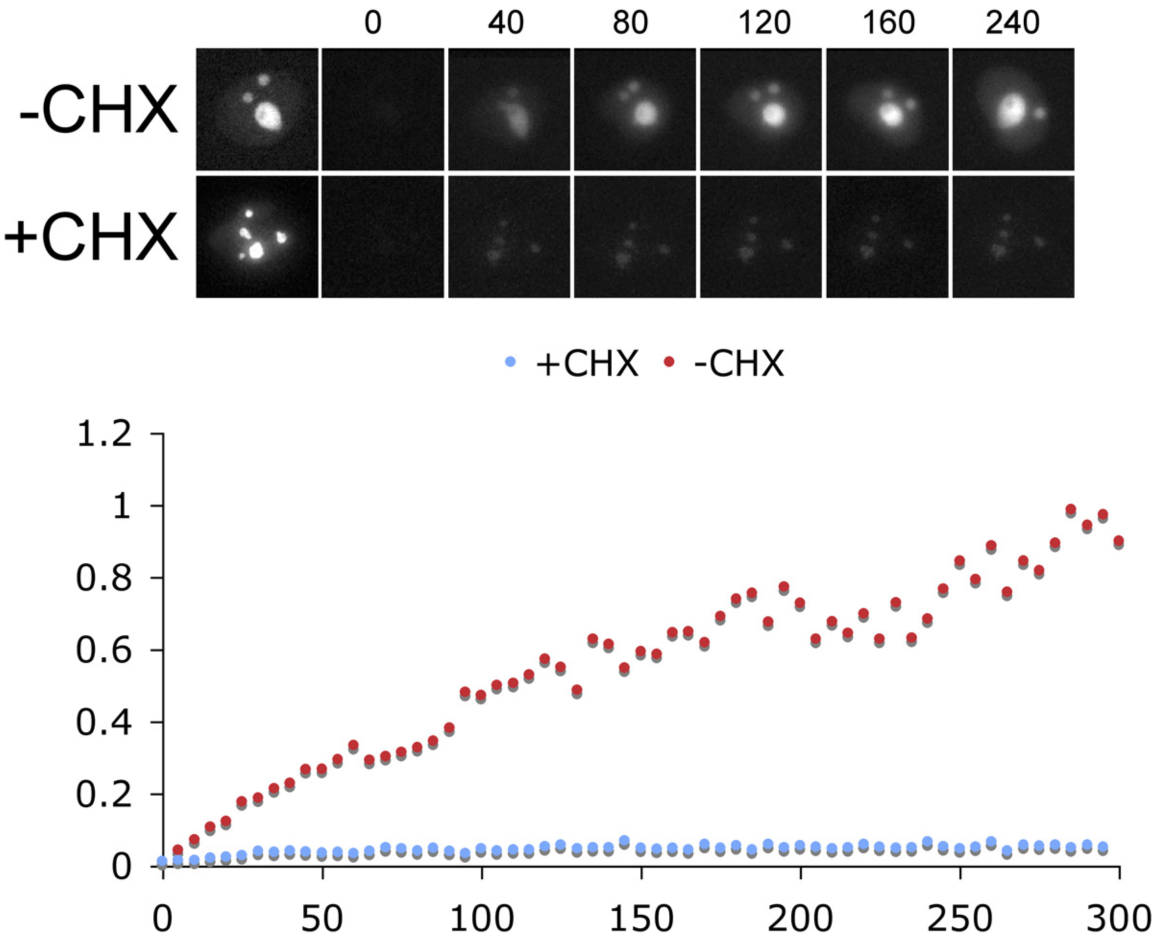

Figure S2. Recovery of Fluorescence after Photobleaching Was Abolished by Pretreatment of Cells with Cycloheximide

Fluorescence signal of a HeLa RPS6-GFP cell was photobleached, and the recovery of GFP fluorescence was measured for 300 min (red). In a separate experiment, cycloheximide was added to the medium to a final concentration of 100 μg/ml. One hour after treatment, the GFP signal of a HeLa $^{RPS6\text{-}GFP}$ cell was photobleached and the recovery of GFP fluorescence was measured for 300 min (blue).

Mass-Spectrometric Analysis and Quantitation of Isotope-Labeled Peptides

Mass-spectrometric analysis was performed by liquid chromatography (Agilent HP1100) combined with tandem MS (LC MS/MS) with a linear ion-trap Fourier-transform ion-cyclotron-resonance mass spectrometer (LTQ-FT-ICR, Thermo-Finnigan). The instrument was operated in the data-dependent mode so that it would automatically switch among MS, MS^2, and MS^3 acquisition. Precursor ion spectra (m/z 300−1500, R = 25,000 at m/z 400) were acquired by Fourier-transform ion-cyclotron resonance (FT ICR) after ion accumulation to a target value of 10,000,000 in the linear ion trap. The three most intense ions were sequentially isolated for accurate mass measurements by selected ion monitoring (SIM) scans (17–25 Da mass window, R = 50,000, and a target accumulation value of 50,000). The ions were simultaneously fragmented in the linear ion trap with a normalized collision energy setting of 27%–30% and a target value of 5000 for MS^2 and MS^3 spectra. Ion-selection thresholds were as follows: 500 counts for MS^2 and 50 counts for MS^3. An activation q = 0.25 and activation time of 30 ms was applied in both MS^2 and MS^3 acquisitions. Former target ions selected for MS^2 were dynamically excluded for 30 s. The mass-spectrometric conditions were as follows: spray voltage, 2.4 kV; no sheath and auxiliary gas flow; ion-transfer tube temperature, 100°C; collision gas pressure, 1.3 mTorr. Peak lists were extracted from the ms data with in-house-developed software (DTA supercharge) and used for protein identification in the international protein-index database with the Mascot program (Matrix Science). The searches were performed with strict trypsin specificity and a mass tolerance of 3 ppm for precursor ions and 0.6 Da for fragment ions and by allowing for two missed cleavages and with the following modifications: fixed modification: carbamidomethylation of cysteine; variable modification: oxidation of methionine, protein N-acetylation, and SILAC amino acids. The peptide ratios were calculated as the $^{13}C_6/^{12}C_6$ peak area of each single-scan mass spectrum and averaged for all arginine- and lysine-containing peptides for each protein. MS Quant, an in-house-developed software program, was used to evaluate the certainty in peptide identification and in peptide-abundance ratio. The fraction of isotope incorporation for time-course-labeled peptides were calculated as 1/([1/ratio] + 1).

Microscopy

Cells were cultured in glass-bottomed dishes (WILCO; Intracel). Before imaging, growth medium was replaced with Phenol red-free CO_2-independent medium (Invitrogen). Cells were found to grow and proliferate normally in this medium for at least 48 hr. In all experiments, an Olympus 40× oil immersion lens (NA 1.35) was used. DIC imaging was done with the appropriate prism insert (Olympus).

Photobleaching was performed with 100% laser power for the duration of 0.05 s. The subcellular locations of bleaching events were selected on the basis of bright-field DIC microscopy. In some experiments, the entire nucleus, cytoplasm, or cell was outlined with the "polygon" function, and photobleaching then was performed with the "pattern spacing" of five. In all experiments, except those reported in Figure 7 and Figure S6, ten optical sections (1.2 μm each) were collected at every time points, with an exposure time of 0.1 s for imaging GFP fluorescence.

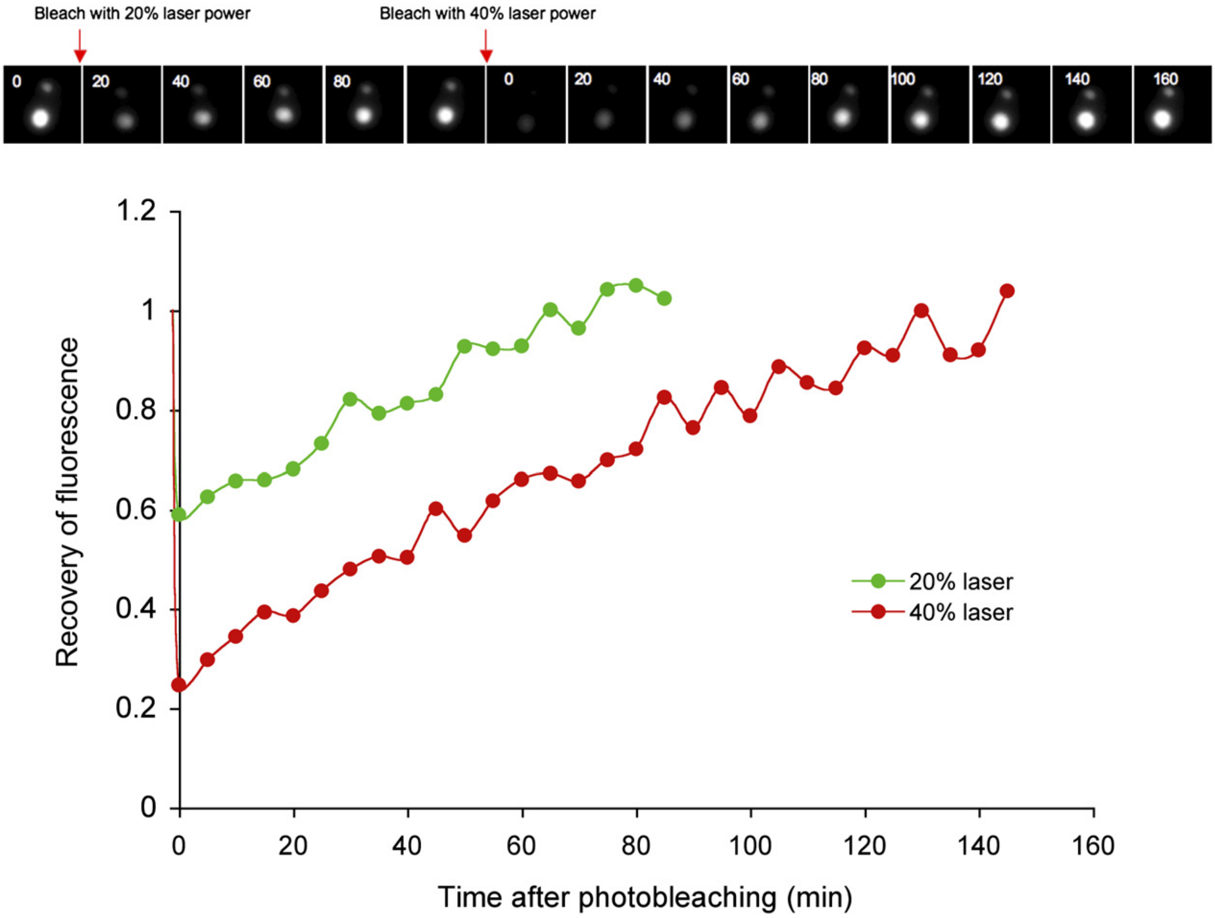

Figure S3. Rate of Recovery of Fluorescence after Photobleaching Was Independent of the Laser Power Used

Fluorescence signal of a HeLa RPS6-GFP cell was photobleached with 20% of full laser power (488 nm laser), and the recovery of GFP fluorescence was measured for 80 min. Then, the GFP signal of this cell was photobleached again, this time with the laser power set at 40%. The recovery of GFP fluorescence was measured for 160 min.

For measurement of the rate of nucleolar import of RPL27-GFP (Figure 1), the nuclear GFP fluorescence of HeLa $^{RPL27\text{-}GFP}$ cells was photobleached and the recovery of fluorescence in the nucleolus and cytoplasm was monitored for 100 min. Then, cycloheximide was added to the culture medium to a final concentration of 100 μg/ml. After 5 min, the nuclear GFP fluorescence of the same cells was bleached again and the recovery of fluorescence in the nucleolus and cytoplasm was monitored for another 100 min.

For data shown in Figure 7 and Figure S6, HeLa $^{RPL27\text{-}GFP}$ cells were photobleached with the "photokinetic experiment" function of the DeltaVision Spectris microscope. In brief, small regions in the cells were photobleached with a 488 nm laser (100% laser power for 0.05 s), and timelapse sequences of single optical sections were collected, with an exposure time of 0.1 s for each image. SoftWorX software (Applied Precision) was used for image acquisition and data deconvolution. In these FRAP experiments, the X, Y, and Z positions of five (Figure 7) or ten (Figure S6) randomly chosen HeLa$^{RPL27\text{-}GFP}$ cells were marked, with the "mark position" function of Deltavision. These cells were analyzed by FRAP, and this was followed by the incubation with drugs or DMSO (as indicated in the figure legends). Then, FRAP was performed at the same subcellular locations of the same marked cells.

The 3D datasets were rendered into 2D projections with the "sum" algorithm. The fluorescence intensities in different subcellular locations were quantitated with the software Advanced Image Data Analyzer (Aida) version 3.27 (Raytest). Data reported in Figure 7 and Figure S6, including the calculated recovery half times and mobilities, were generated by the built-in "photokinetic data analysis" function of Softworx.

Other Procedures

Characterization of cell-cycle-stage distribution by flow cytometry was performed as described [S3]. Fractionation of cytoplasmic ribosomal subunits and polysomes by ultracentrifugation and sucrose gradient was performed as described [S4].

Supplemental References

S1. Thomas, F., and Kutay, U. (2003). Biogenesis and nuclear export of ribosomal subunits in higher eukaryotes depend on the CRM1 export pathway. J. Cell Sci. *116*, 2409–2419.

S2. Dai, M.S., and Lu, H. (2004). Inhibition of MDM2-mediated p53 ubiquitination and degradation by ribosomal protein L5. J. Biol. Chem. *279*, 44475–44482.

S3. Trinkle-Mulcahy, L., Sleeman, J.E., and Lamond, A.I. (2001). Dynamic targeting of protein phosphatase 1 within the nuclei of living mammalian cells. J. Cell Sci. *114*, 4219–4228.

S4. Tyzack, J.K., Wang, X., Belsham, G.J., and Proud, C.G. (2000). ABC50 interacts with eukaryotic initiation factor 2 and associates with the ribosome in an ATP-dependent manner. J. Biol. Chem. *275*, 34131–34139.

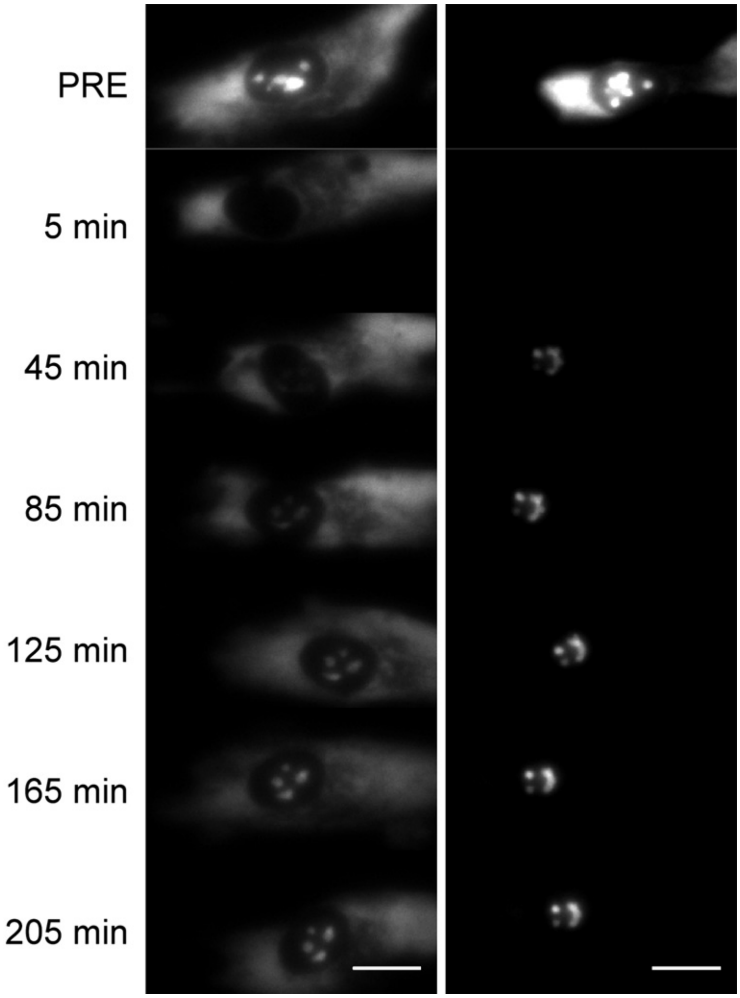

Figure S4. RPL27-GFP Nuclear Import and Export in Human Retinal Pigmented Epithelial Cells

Left panels show that the nuclear fluorescence of a human retinal pigmented epithelial cell transiently expressing RPL27-GFP was photobleached, and the recovery of fluorescence was measured for 205 min. Right panels show that the fluorescence of an entire human retinal pigmented epithelial cell expressing RPL27-GFP was photobleached, and the recovery of fluorescence was measured for 205 min. Scale bars represent 5 μm.

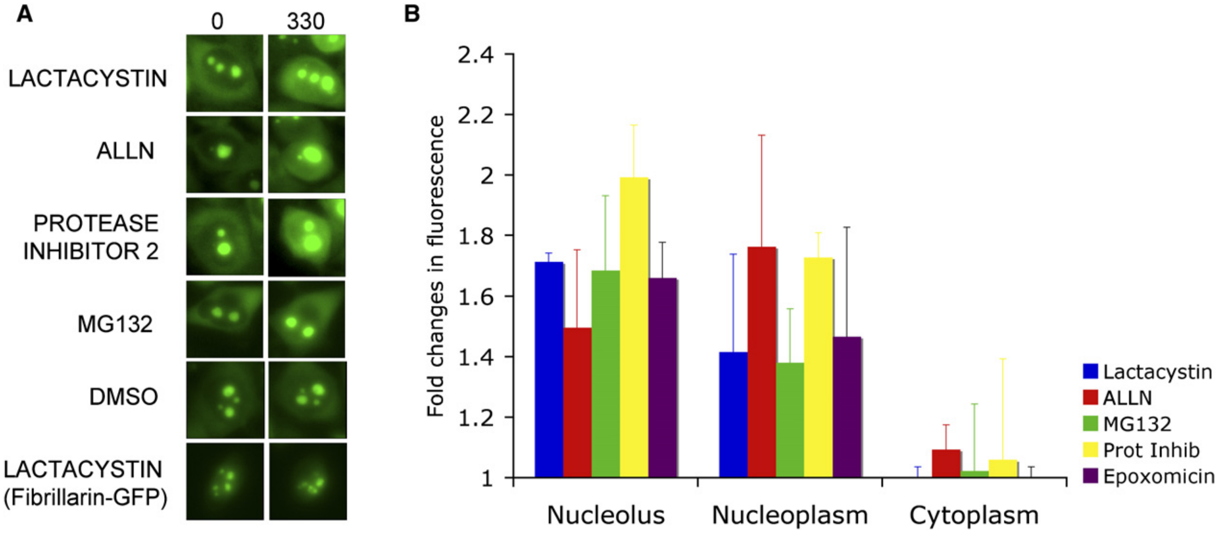

Figure S5. Ribosomal Protein Accumulates in the Nucleus but Not the Cytoplasm on Proteasomal Inhibition

(A) HeLa $^{RPL27\text{-}GFP}$ cells before (left panels) and 330 min after (right panels) treatment with lactacystin, ALLN, protease inhibitor 2, MG132, or DMSO alone. All inhibitors were used at 25 μM, diluted from 25 mM stocks in DMSO. The lowest panels show HeLa$^{Fibrillarin\text{-}GFP}$ cells treated with 25 μM MG132.

(B) Quantitation of GFP fluorescence in the nucleolus, nucleoplasm, and cytoplasm of HeLa$^{RPL27\text{-}GFP}$ cells after treatment with proteasome inhibitors (relative to the intensity in the same regions prior to treatment). Average ± SD of five cells is shown for each treatment.

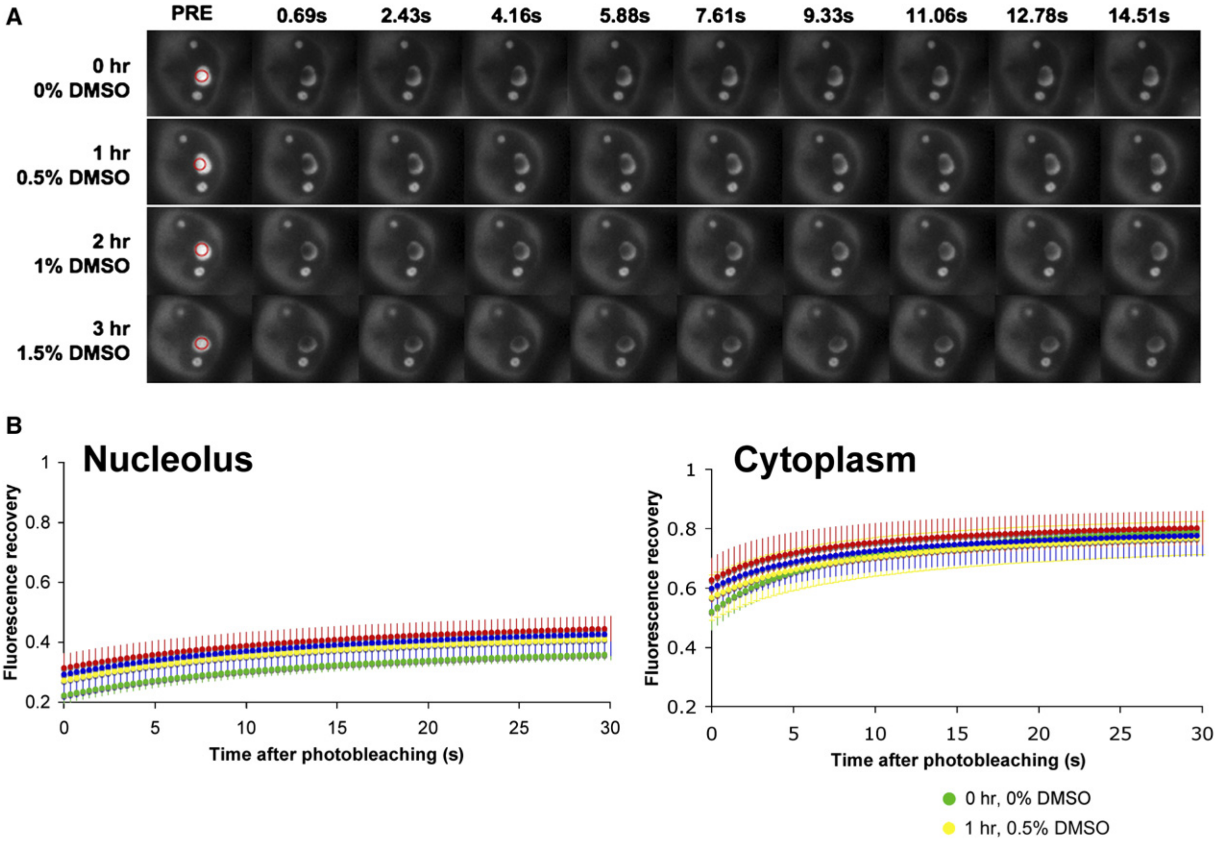

Figure S6. Repeated Photobleaching in the Same Spots Does Not Alter the Fluorescence Recovery Rate of RPL27-GFP

(A) A region in the nucleolus of a HeLa $^{RPL27-GFP}$ cell was photobleached, and the recovery of fluorescence was measured. A total of 0.5% (v/v) DMSO was added to the medium. After 1 hr, the same region in the same nucleolus of the cell was photobleached again and the recovery of fluorescence was measured. Then, an additional 0.5% (v/v) DMSO was added (making the final DMSO concentration 1%), and the FRAP measurement was made in the same region again 1 hr later. An additional 0.5% DMSO was added, and FRAP was repeated once more on the same region.

(B) The quantitation of the repeated FRAP measurements in the nucleolus (left) and cytoplasm (right), showing average ± SD of ten cells.

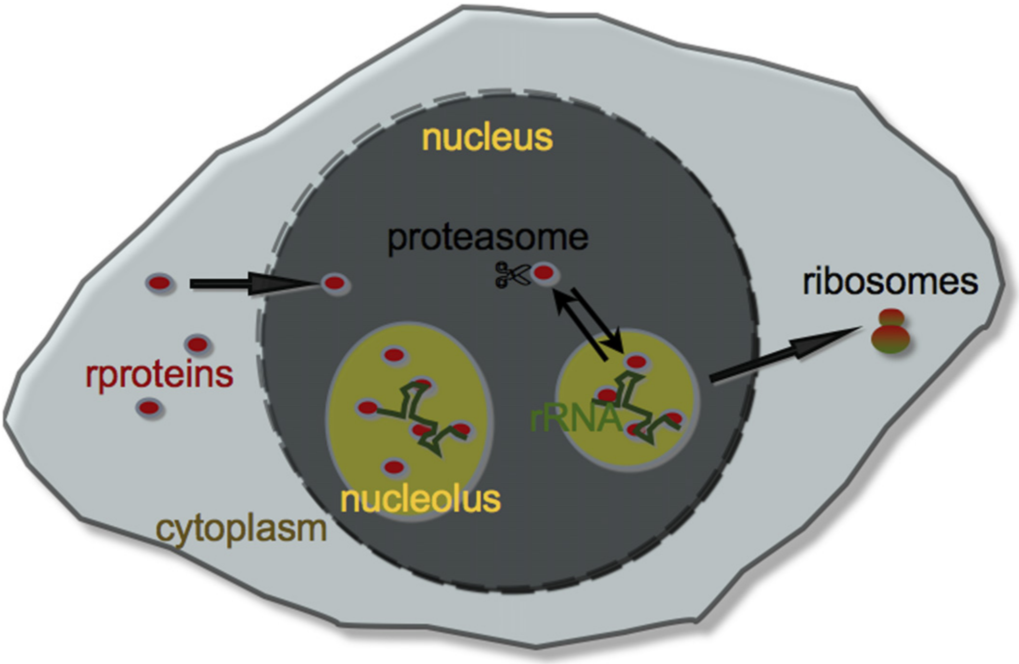

Figure S7. Model of rprotein Dynamics

Newly synthesized rproteins are rapidly imported into the nucleus, where they shuttle between the nucleolus and nucleoplasm. Some rproteins bind to rRNA in the nucleolus and are assembled into ribosomes, which are exported to the cytoplasm. However, a proportion of rproteins remain unbound, which varies according to the level of rRNA synthesis. The unbound pool shuttles between the nucleolus and nucleoplasm and is exposed to proteasomal degradation when it enters the nucleoplasm.
